# 1516. High Incidence of Newly Infected Syphilis, Hepatitis B and C Virus among People Living with HIV Who Have Reached U=U

**DOI:** 10.1093/ofid/ofad500.1351

**Published:** 2023-11-27

**Authors:** lingyun Ge, Qiaoli Peng, Yun He, Jiaye Liu

**Affiliations:** Medical School, Shenzhen University, Shenzhen, Guangdong, China; The University of Hong Kong, Shenzhen, Guangdong, China; Shenzhen Third People’s Hospital, Shenzhen, Guangdong, China; Shenzhen University Health Science Center, Shenzhen, Guangdong, China

## Abstract

**Background:**

The concept of “Undetectable equals Untransmittable (U=U)” has been widely accepted. However the risk of acquiring other sexually transmitted diseases (STDs) such as syphilis (TP), hepatitis B virus (HBV) and hepatitis C virus (HCV) in people living with HIV (PLWH) in the context of “U=U” remains unclear. This study evaluated the incidence of newly STDs in PLWH in Shenzhen, China.

**Methods:**

128 virally suppressed PLWH were investigated from March 21 to April 7 2023 at the Third People’s Hospital of Shenzhen. Information on sexual behavior, the awareness rate of U=U and clinical records were collected. Results of HBSAg, HCV RNA, TP antibody before ART(calculating baseline prevalence) and in follow-up(calculating incidence) were collected. The STD incidences among various subgroups were compared.

**Results:**

At baseline, the prevalence of TP, HBV and HCV were 29.7%(38/128), 15.6%(20/128), and 1.56%(2/128). 78(60.9%) PLWH were not coinfected with any STDs, of which were male with a median age of 36.8 years (IQR 29, 42) and median duration of ART 4.9 years (IQR 2.3, 7), 75(96.2%) were men who have sex with men(MSM). Twelve TP, six HBV and five HCV new infections occurred over 382.9 person-years; the corresponding incidences were 3.13/100 person-years, 1.57/100 person-years and 1.31/100 person-years, respectively. One participant had TP/HBV co-infection, one had HBV/HCV, and one had TP/HCV. Surprisingly, all six newly HBV-infected PWLH received a TDF/TAF+XTC-based regimen. Among the 45 participants who had reached the awareness of U=U and 33 who had not, the new TP incidence were 2.35/100 and 4.11/100 person-years, the new HBV incidence were 1.88/100 and 1.76/100 person-yearsand, the new HCV incidence were 1.88/100 and 1.17/100 person-years(p > 0.05), respectively.

**Conclusion:**

The high incidence of newly acquired TP, HBV and HCV infection after reaching U=U highlights the necessity of STD screening and prevention in viral suppression population in PWLH.

**Disclosures:**

**All Authors**: No reported disclosures

